# Intervention on Externalizing Problems of Undercontrolled Personality Types in Primary School Students

**DOI:** 10.3389/fpsyg.2020.01233

**Published:** 2020-06-23

**Authors:** Yongjin Yu, Lizhu Yang, Yan Sun, Chenhui Jin, Ying Zhang

**Affiliations:** ^1^School of Philosophy and Sociology, Jilin University, Changchun, China; ^2^College of Psychology, Liaoning Normal University, Dalian, China; ^3^Bayi Primary School, Dalian, China; ^4^Shidao Street Primary School, Dalian, China

**Keywords:** personality, undercontrolled types, primary school students, externalizing problems, intervention

## Abstract

Three personality types (resilient, overcontrolled, and undercontrolled) have been repeatedly verified across different languages and cultures, different personality models, and different stages of development. Undercontrollers are socially maladapted types with high impulsivity and low self-control. Research shows they are at risk for externalizing problems, such as aggressiveness, impulsivity, and antisocial behavior. The aim of this study was to develop an intervention to reduce externalizing problems of undercontrolled personality types in primary school students. Participants were 69 undercontrolled primary school students from two primary schools in North China. The experimental group underwent 14 weeks of systematic experiential mental health activities, while the control group performed typical daily classroom activities. Personality and externalizing problem behaviors were measured before the intervention, at the end of the intervention, and 4 months post-intervention. The results showed that the intervention significantly reduced the level of externalizing problems of undercontrolled primary school students. The effects of the intervention were maintained at the 4-month follow-up. This study provides some reference and suggestions on how to intervene in the externalizing problem behaviors of undercontrolled primary school students.

## Introduction

A significant body of evidence has revealed that personality not only affects individuals’ current academic achievements, health, and peer relationships but also has a positive predictive effect on future life and income (Leikas and Salmela-Aro, 2014; Reitz et al., 2014; Gray and Pinchot, 2018; Jonason et al., 2018; Stajkovic et al., 2018). Personality has been examined from person- and variable-centered perspectives in extant literature (Donnellan and Robins, 2010). The variable-centered approach to personality is primarily reflected in studies on personality dimensions or traits (Bergman and Magnusson, 1997). The widely employed five-factor model (FFM) comprises the personality dimensions of openness, conscientiousness, extraversion, agreeableness, and emotional stability (John et al., 2008). However, two limitations are presented from a variable-centered perspective. First, the fact that individual consistency scores vary among individuals is ignored. Thus, the variable-centered approach disregards inter-individual differences in individual consistency (Asendorpf, 2015). The second limitation involves the disregard for the organization of traits within individuals (Donnellan and Robins, 2010). Personality may be defined as a combination of different traits; these traits do not function in isolation from each other within a person but function as a coordinated system of traits (Digman, 1990; McCrae and John, 1992). The person-centered approach seeks to avoid the limitations of the trait approach by highlighting individuals and the organization of traits in individuals (Grumm and Collani, 2009). Further, it emphasizes that personality type can explain the interaction between traits and the integrity of personality (Donnellan and Robins, 2010). Generally, there are three personality types (resilient, undercontrolled, and overcontrolled) that present different combinations of traits (Asendorpf et al., 2001; Rosenström and Jokela, 2017). Initially, the interpretation of the three types of connotations was based primarily on the combination of two dimensions, namely, ego-control and ego-resilience (Block and Block, 1980). The resilient personality type is a combination of high ego-control and high ego-resilience. The undercontrolled personality type is a combination of low ego-control and low ego-resilience. The overcontrolled personality type is a combination of high ego-control and low ego-resilience. Subsequently, according to the results of several studies, the five personality dimensions in FFM can be combined with each other to form the same three personality types (Robins et al., 1996; Asendorpf and Van Aken, 1999; Asendorpf et al., 2001; Yang and Ma, 2014; Rosenström and Jokela, 2017). Whereas the five dimensions of resilient types are all above average, the five dimensions of undercontrolled types are all below average. In contrast, the overcontrolled personality type scores below average for only extraversion and emotional stability (Van Aken and Semon Dubas, 2004; Grumm and Collani, 2009; Yang and Ma, 2014). These three personality types have been verified across different cultures and ages repeatedly (Alessandri et al., 2014; Specht et al., 2014; Yang and Ma, 2014).

### Undercontrolled Personality Types and Externalizing Problems

Undercontrollers are socially maladaptive, with high impulsivity and an inability to adjust their self-control in relation to situational demands (Block and Block, 1980; Letzring et al., 2005). Undercontrollers are at risk of suffering academic and externalizing problem behaviors (Yu et al., 2015; Bohane et al., 2017). Externalizing problem behavior refers to problems that are evident in children’s outward behavior, such as antisocial behavior, aggression, impulsivity, and delinquency (Achenbach et al., 2016). Previous research has shown that undercontrolled primary school students have a high level of externalizing problem behaviors, such as confrontation with teachers, disobedience, abusing, or fighting with classmates, many small actions during the learning process, distraction, disturbing the class, restlessness, impulsivity, stealing, and lying (Devos et al., 2012; Isler et al., 2016; Bohane et al., 2017). These behaviors not only thwart the teacher’s lesson plan and disrupt class activities but also affect undercontrolled students’ social adaptation, personality development, and mental health. Therefore, it is necessary to improve the externalizing problem behaviors of undercontrolled primary school students.

### Relationships Between Personality Traits and Externalizing Problems

Evidence from various studies has suggested a certain degree of specificity in the relationship between personality traits and behavior problems (Kotov et al., 2010; Mezquita et al., 2015). For instance in a meta-analysis, MacLaren et al. (2011) found that people with low conscientiousness and low agreeableness exhibited externalizing behaviors (i.e., aggressive behavior and antisocial behavior). Several studies have demonstrated that individuals with low scores on conscientiousness and agreeableness exhibited high levels of externalizing behavior problems (Tackett, 2006; De Haan et al., 2010; Prinzie et al., 2010; Slobodskaya and Akhmetova, 2010; Stoltz et al., 2013; Van den Akker et al., 2013). It can be seen that the personality dimensions of conscientiousness and agreeableness are the strongest predictors of externalizing problems behaviors (Jones et al., 2011; Frick and Ray, 2015). Individuals with the undercontrolled personality type are most often observed to have lower than average conscientiousness and agreeableness (Yang and Ma, 2014; Rosenström and Jokela, 2017). Thus, it is not difficult to understand why undercontrollers exhibit a high level of externalizing behavior problems.

### Personality-Targeted Interventions

Personality development is influenced by social and environmental factors, thus suggesting that personality intervention is feasible (Donnellan and Robins, 2010; Zou et al., 2016). Personality-targeted interventions may effectively change the recurrence of externalizing problems a long time after intervention (O’Leary-Barrett et al., 2016). This kind of intervention has realized the goal of reducing externalizing problems by changing personality traits (Castellanosryan et al., 2016). For example, O’Leary-Barrett et al. (2013) tested the effects of a brief, personality-targeted intervention on internalizing and externalizing symptoms. The participants, 1024 students in 19 London schools, in experimental and control conditions completed follow-up questionnaires at 6-monthly intervals for 2 years to assess the long-term impact of the intervention. This study selected personality traits that had a risky impact on physical and mental health as the target of the intervention. The results revealed that after the intervention, the experimental group’s internalizing and externalizing behavior problems decreased significantly. Furthermore, personality traits can be employed as not only direct intervention objects to change behavior problems but also indicators for evaluating the effects of behavior problem interventions (Roberts et al., 2017). A study on the intervention of subjects with anxiety problems revealed that the neuroticism and extraversion of the subjects in the experimental group changed as their anxiety levels diminished (Carl et al., 2014).

The study outlined above was variable-centered and was only able to examine the average level change of a certain personality trait in the group in isolation. The study of personality types from a person-centered perspective supplements the study of personality traits from a variable-centered perspective (Vollrath and Torgersen, 2008). A person-centered perspective is necessary, with the unit of analysis being the person, not a trait, and the organization of many traits can be studied. Moreover, studying personality types from a person-centered perspective is beneficial in interventions in groups with similar behavior patterns.

### The Present Study

The current study aimed to develop and test an intervention for primary school children, designed to reduce externalizing problem behaviors displayed by individuals characterized as undercontrollers. Based on earlier work, we hypothesized that (1) the externalizing problem behavior of undercontrolled primary school students would be reduced by enhancing the personality traits of conscientiousness and agreeableness; (2) 4 months after the end of the intervention, the effects of the intervention would be maintained.

This study is different from previous studies in several ways. First, in order to understand the specific behavior of undercontrolled primary school students’ externalization problems, we interviewed primary school teachers. Second, we also referred to previous research and designed intervention curricula suitable for 10-year-old children. Third, regarding the intervention of externalizing problem behaviors, a large number of previous studies have focused on specific personality traits. This study focused on personality types and intervened on a certain type with problem behaviors. Fourth, we designed intervention curricula based on personality traits related to the externalizing problems, with the aim of improving the undercontrollers’ problem behaviors. It also has a clear intervention purpose and is easily administered. Finally, we examined the effects of the intervention curricula four months later.

## Materials and Methods

### Participants

We selected two primary schools in North China to issue the questionnaire. The questionnaire was used to evaluate 234 primary school students (126 male and 108 female) in two schools, with an average age of 10 years old. Latent class analysis (LCA) was used to classify the personality type among 234 primary school students. The intervention is normally influenced by some variables. Thus, we intervened in the natural class where undercontrolled type students were located. This study was approved by the local ethics committees of Liaoning Normal University. Written informed consent had been obtained from the parents/legal guardians of all participants. All participants were volunteered to join the experiments, and informed consents were signed by their legal guardians.

### Instruments

#### Personality Inventory for Primary School Student

Teacher rated their students’ personality on the Personality Inventory for Primary School Student (Zhang, 2011). The personality was measured using Zhang’s Chinese FFM, which was developed based on the original FFM. This personality inventory includes five dimensions, namely, extraversion, agreeableness, conscientiousness, emotional stability, and intelligence. This inventory includes 62 items. All items were rated on a five-point Likert scale from 1 (very inaccurate) to 5 (very accurate). The Cronbach’s alphas and the omegas of scale reliability and validity are presented in Table 1.

**TABLE 1:** The descriptive data of the scale.

	Mean (standard deviation)	Cronbach’s α	McDonald’s ω
Extraversion	3.64 (0.677)	0.928	0.928
Agreeableness	3.79 (0.661)	0.939	0.945
Conscientiousness	3.63 (0.745)	0.947	0.955
Emotional stability	3.74 (0.974)	0.922	0.923
Intelligence	3.54 (0.784)	0.965	0.966
Externalizing problems	1.39 (0.246)	0.920	0.924

#### Child Problem Behavior

Parents rated their child’s problem behavior with the Chinese version of the Child Behavior Checklist (CBCL; Achenbach, 1991). The CBCL contains 120 items covering behavioral or emotional problems. The response format is 0 (not true) through 2 (very true or often true). Two broadband factors can be derived from the CBCL scales: internalizing problem behavior and externalizing problem behavior. Externalizing problem behavior includes items related to aggression, hyperactivity, and delinquency. The Cronbach’s alphas and the omegas of scale reliability and validity are presented in Table 1.

### Design and Procedures

#### Intervention Goals

Based on previous research, we selected the dimensions of conscientiousness and agreeableness for personality-targeted interventions. These two personality dimensions are also closely related to externalizing problems in this study (see Supplementary Figure S1). Under the theoretical framework of the Chinese FFM, conscientiousness and agreeableness contain seven facets: concentration and responsibility; self-control; aggression and resistance; planning and orderliness (conscientiousness); honesty and shame; sympathy and altruism; and gregariousness and courtesy (agreeableness) (Zhang, 2011). In order to effectively reduce the level of externalizing problems of undercontrolled primary school students, the intervention goals were designed around the specific seven facets under the two personality dimensions that have a significant relationship with externalizing problems. Based on the implications of the seven facets, we developed a description questionnaire for intervention goals of undercontrolled primary school students’ problem behaviors. The description questionnaire for intervention goals of undercontrolled primary school students’ problem behaviors was filled out by parents and teachers. Parents and teachers filled in children’s actual problem behaviors based on the connotations of specific facets (see Table 2). By sorting and analyzing the specific problem behaviors described by teachers and parents in the description questionnaire for intervention goals of undercontrolled primary school students’ problem behaviors, we obtained a total frequency of 803 instances of the same problem behaviors. We used coding method to summarize and describe the sentences described by parents and teachers. Similar sentences fall into one category. There are three behavioral categories under each facet. According to the frequency of problem behavior performance, we summarized two intervention goals for each facet, as shown in Table 2.

**TABLE 2:** Problem behaviors, intervention goals, and activity programs for undercontrolled primary school students.

Facets	Connotations	Problem behavior exhibited	Intervention goals	Activity programs
CR	Attentive, meticulous, and dedicated to class activities.	They are not attentive, careless in class, lack a sense of responsibility and participation in class activities, lack of dedication and sense of collective honor and shirk responsibility after making mistakes.	1. Listen carefully in class, pay attention and study, and do things undisturbed.	Focus my attention. Attention training.
			2. Dare to admit mistakes, do not shirk responsibility, and have a sense of responsibility for class things.	Have a sense of responsibility. Happy race.
SC	Be able to control your words and deeds and be persistent in activities and accomplish them.	They cannot control their mouths and speak casually; they are impulsive in doing things; they cannot control their words and deeds; they do not obey rules and violate discipline; when they encounter difficulties in studying or doing things, they quit halfway, shrink back, and cannot stick to the end.	1. In the case of unsupervised control of self-expression and behavior in line with the code of conduct.	Obey the rules. I want to control myself.
			2. Consciously study, control the impulsive mood, and continue to work hard when encountering learning difficulties.	Perseverance keeps me progress. Learn to wait.
PO	In the activity, you can set goals, set steps, and complete tasks in an orderly manner.	They do not have the concept of time, cannot set reasonable goals and plans by themselves, and can only complete the prescribed tasks under supervision; books and schoolbags are not arranged neatly and randomly; they have no clue and no steps and cannot complete the tasks in an orderly.	1. Be able to set goals with help and complete plans with the supervision of others.	Plan to lead learning. How to spend weekends.
			2. Set goals for yourself and work hard to implement them as planned in an orderly manner.	My time is up to me. Methodically.
AR	Weak self-control, hostile attitude toward others, and poor handling of conflicts.	It is easy to fight and verbally abuse when conflicting with others; damage others’ objects and be hostile to others; cause trouble for no reason; cause rebellion, resistance, and confront teachers and parents, and do not accept criticism and education.	1. Do not attack others or reduce the number of aggressive behaviors.	Methods other than attack. To turn war into silk.
			2. Learn to communicate with teachers and parents equally and overcome rebellious psychology.	Communication strongman. Please help me.
SA	Take the initiative to help and comfort others and show compassion and sympathize with others.	Lacks compassion and love, does not care about others, does not help others, does not consider parents and teachers, ignores the feelings of others, considers himself/herself in advance, is more selfish and self-centered.	1. Take the initiative to help others and do things that are beneficial to others.	Friendship for the disabled. Caring for others.
			2. The help in behavior gradually develops a spiritual resonance and can care and sympathize with others.	Gifts of roses. Compare heart to heart
GC	Well integrated into the group, good communication with peers, effective cooperation with others in learning activities, and common goals.	They are independent and not good at communicating with others, have few friends, cannot get along well with others, and do not want to share; they do not have strong sense of cooperation and like to work alone; they cannot complete tasks together with groups.	1. Be enthusiastic, proactive, friendly, and polite when dealing with others.	Know etiquette and be polite. Take the initiative.
			2. Cooperate with others in group activities and be more integrated into class groups.	Cooperation skill. Cooperative minibus.
HS	Dealing with people in good faith, not lying, re-commitment, and shame when doing things that are against moral norms.	Love lying, not sincere, cannot do what they say, do not keep promise, lack of understanding of moral norms, do wrong, do not know shame.	1. After doing something wrong, admit it, treat others with sincerity and do not lie.	Be an honest child. Lying and honesty.
			2. Promise that others can do what they say and keep their promises.	Trustworthy me. Speak with faith.

*PS: CR, concentration and responsibility; SC, self-control; AR, aggression and resistance; PO, planning and orderliness; HS, honesty and shame; SA, sympathy and altruism; GC, gregariousness and courtesy.*

#### Intervention Curricula

The undercontrolled children engaged in a 14-week intervention in natural classes of twice a week for 45 min each time. We chose system experiential mental health activities as the best carrier. These mental health education activities were mainly carried out in an experiential way in accordance with system theory. According to system theory, interventions should occur in the environment where the child with the problem behavior is located. By locating the intervention in the child’s environment, good peer relationships are established, which helps to reduce problem behaviors (Bowen et al., 2010). For primary school students, the environment mainly refers to the school. This is also consistent with the theory of group socialization development, emphasizing the role of peer groups in the process of children’s socialization. Peer groups promote the development of children’s sociality and individuality through the assimilation and alienation mechanism. Therefore, we designed some interactive games to establish and strengthen good peer relationships. According to the flow experience theory, when an individual has a strong interest in an activity, he or she will focus more on the things they pay attention to and they will experience emotions such as enjoyment, enrichment, and contentment. In this state of motivation and mental pleasure, an individual can maximize their potential to acquire certain knowledge or skills (Csikszentmihalyi, 1998). Leontev (1978) activity theory also emphasizes that activities should value children’s learning interests and their direct experiences. Through physical activities, children experience and gain a rational and deep understanding through physical actions in a relaxed state. Through actual experience to self-summary, children continue to internalize the positive qualities in activities to achieve healthy development. Therefore, in order to promote the alleviation of problem behaviors of undercontrolled students, students should be allowed to participate in and experience activities. This can transform the intrinsic educational value of the activities into the actual development level of children.

Guided by the intervention goals, we designed a 14-week intervention curriculum. First, for each of the seven facets of conscientiousness and agreeableness, we designed two intervention goals (see Table 2). Two activities were designed for each intervention goal. Thus, there were 28 activities designed for seven facets. For example, under the facet of self-control, in order to achieve the goal of “Controlling behaviors in accordance with the code of conduct without supervision,” we designed two activities: “Obey the rules” and “I want to control myself.” In the “I want to control myself” activity, students were required to pay attention to the teacher’s instructions. After the teacher gave the instruction, the students quickly made the opposite action to the instruction. For example, if the teacher says to turn left, the students should turn right. In this activity, Go/No Go technology was used to improve the impulse suppression of undercontrollers and control their own words and deeds in accordance with the code of conduct. Second, the activity program was based on the developmental characteristics of primary school students. For example, around the age of 10 is the critical period for the development of prosocial behavior and it is also the initial period for the formation of peer groups (Liu, 2013). At this stage, establishing good peer relationships is conducive to the development of prosocial behavior (Pan and Cao, 2009). Therefore, we designed the “Blind and Crutches” activity, wherein students experience the joy of helping each other by random matching, promoting friendship, and better peer exchanges and training children to show more helping behavior. We asked experienced teachers to comment on the intervention curriculum and then modified and improved the activity programs based on their opinions. The final intervention curriculum is presented in Table 2.

### Statistical Analysis

The SPSS 20.0 was used to conduct descriptive statistics and analysis of variance. The Mplus 7.4 was used to conduct LCA. All data were treated with a statistical significance level of *p* < 0.05. LCA and its fit indices such as AIC, BIC, adjusted BIC, entropy, LMR, and BLRT output by Mplus were used to identify primary school students’ personality types (Muthén and Muthén, 2000; Nylund et al., 2007). First, the best model has lower AIC, BIC, and aBIC (Schwarz, 1978). Second, the range of entropy is between 0.00 and 1.00. The higher the value of entropy, the higher the classification accuracy (Hix-Small et al., 2004). Third, if the *p*-values for LMR and BLRT are significant for a k class model, the k class model will significantly improve over a k-1 class model (Asparouhov and Muthén, 2012). Fourth, if some types in a k class model already appear in a k-1 class model, the k-1 class model will be selected according to the principle of model simplicity (Muthén and Muthén, 2000).

The experimental and control groups were selected from the undercontrolled primary school students. The personality and externalizing problems of the children from all groups were tested at three time points: before the intervention (T1); 14 weeks later, immediately following the intervention (T2), and 4 months after of the end of the intervention (T3). We analyzed the effectiveness of the intervention by means of repeated measurement ANCOVAs (repeated measurements = post-test, and follow-ups after 4 months; independent variable = the activity program; covariate = pre-test score). In the control of unrelated variables, we took the following measures. First, we applied a homogeneity test on the pre-test group to ensure both groups’ developmental level of personality and externalizing problems. Second, while the experimental group underwent intervention in a mental health class, the children in the control group engaged in mental health lessons prescribed by normal teaching. To prevent students, parents, and teachers from guessing the experimental expectations, this study intervened in the entire class of undercontrolled individuals. During each assessment, parents and teachers assess each student in the class without knowing which class is the experimental one. Additionally, other daily activities remained the same. Uniform requirements were imposed on all teachers, and teachers were not allowed to impose additional activities on the children. Fourth, the intervention and test subjects remained the same throughout the experiment. Fifth, we conducted a survey during the pre-test to ensure that there were no additional relevant interventions outside the primary school environment.

## Results

### Participants

The results show that AIC, BIC, and adjusted BIC decrease monotonically with the increase of classification categories. Entropy is relatively large in three and four classifications, which indicates that the correct rate of personality type classification is higher (see Table 3). Although the *p*-value for LMR is not significant for three-class model and four-class model, other fitting indices are more ideal than two-class model. Wang and Bi (2018) pointed out that the final model should be determined in conjunction with the actual meaning of classification. Statistical indicators only provide a reference for decision-making, and the interpretability of each class should also be considered when determining the best model (Muthén and Muthén, 2000). According to the theory of personality functioning proposed by Block and Block (1980), two-class model can divide the subjects into an adaptive type and a maladaptive type. This class model is too rough. The maladaptive type can be divided into two distinct types (overcontrolled type and undercontrolled type) for three-class model. Undercontrollers are characterized by low ego-control. Overcontrollers are characterized by high ego-control. These two types are located at both ends of ego-control (Block and Block, 1980). According to the actual meaning of classification and the interpretability of each class, we choose three-class model. In intervention studies, the three-class model is more practical than two-class model. In the four-class model, the characteristics of the two types in the middle are overlapping and should be regarded as one type. The three-class model has clearer and concise outlines, and the indicators also meet the criteria for suitability of LCA. According to fit indices and the theoretical construction of previous studies, it is reasonable to divide the personality of primary school students into three classes. This result is consistent with the number of types classified by Ma (2016) for 9254 primary school students in China.

**TABLE 3:** Latent class analysis fitting index of personality dimension of primary school students.

Fit indices	One type	Two types	Three types	Four types
*AIC*	8520.00	8112.18	7849.53	7713.06
*BIC*	8554.55	8167.47	7925.55	7809.81
*aBIC*	8522.86	8116.76	7855.82	7721.06
*Entropy*		0.88	0.90	0.93
*P (LMR)*		<0.001	0.10	0.09
*P (BLRT)*		<0.001	<0.001	<0.001

One-way ANOVA and multiple comparisons are used to describe the characteristics of each personality type (see Table 4). The first personality type are low in most personality dimensions, which is typical of the undercontrolled personality type. The second personality type has the highest scores on all five dimensions, which is typical of the resilient personality type. The third personality type scores low on the extraversion and emotional stability dimensions, which is typical of the overcontrolled type. There are 83 undercontrolled students out of 234 primary school students. In order to control irrelevant variables, this study needs to conduct an intervention research on the natural class where undercontrolled children are located. These 83 undercontrolled children come from seven classes in two schools. Due to the influence of objective factors, we cannot conduct intervention research on all natural classes where 83 undercontrolled children are located. Under the circumstance of ensuring that the number of subjects meets the criterion of the G-power test, 69 undercontrolled students from four classes in two schools are used as research subjects. Two of the classes are from the same school. To maintain the homogeneity of the variables (primary school living environment, daily activities, etc.), we randomly selected one of the two classes from each school for the intervention. In total, 35 undercontrolled type students in two classes from two primary schools formed an experimental group (25 male and 10 female). In the same manner, 34 undercontrolled type students in two classes from two primary schools were selected to form a control group (22 male and 12 female).

**TABLE 4:** Descriptive statistics, analysis of variance, and *post hoc tests* on personality types in five dimensions.

Personality types	Intelligence	Conscientiousness	Extraversion	Agreeableness	Emotional stability
1	38.75 ± 7.538	47.55 ± 8.041	33.36 ± 4.143	41.61 ± 6.074	28.29 ± 6.299
2	66.56 ± 3.943	77.03 ± 4.193	52.91 ± 2.692	64.03 ± 2.024	33.84 ± 7.094
3	52.48 ± 4.962	60.87 ± 6.682	41.22 ± 4.229	50.70 ± 4.089	29.86 ± 8.549
*F*(2, 9251)	286.609***	224.387***	282.858***	272.187***	6.132*
The *post hoc test*	1 < 3 < 2	1 < 3 < 2	1 < 3 < 2	1 < 3 < 2	2 > 1 = 3
*Partial*η^2^*_p_*	0.713	0.660	0.710	0.702	0.050

PS: ***p < 0.001; *p < 0.05; <, significantly lower; =, no significant difference; >, significantly higher.

### The Scores of Personality and Externalizing Problems at Three Points: T1, T2, and T3

The scores of children’s personality and externalizing problems at three points are shown in Table 5. In order to better control the experimental variables, we performed statistics on the pre-test results. The results showed that there was no significant difference in the scores of agreeableness between the experimental group and the control group. The experimental group in the scores of conscientiousness was significantly lower than the control group [*F*(1,67) = 21.19, *p* < 0.001, η^2^*_p_* = 0.24]. The externalizing behavior score of the experimental group was significantly higher than that of the control group [*F*(1,67) = 8.02, *p* = 0.006, η^2^*_p_* = 0.11]. It can be seen that in the scores of conscientiousness and externalizing problems, the two groups are not homogeneous. In a realistic educational situation, in order to respect the authenticity of the research and the feasibility of reality, we allow the two groups of subjects to be heterogeneous.

**TABLE 5:** The personality and externalizing problems data of the experimental group and the control group.

Dimension	Experimental group			Control group		
		
	T1 *M (SD)*	T2 *M (SD)*	T3 *M (SD)*	T1 *M (SD)*	T2 *M (SD)*	T3 *M (SD)*
Conscientiousness	2.73 (0.51)	3.57 (0.92)	3.69 (0.26)	3.19 (0.30)	2.82 (0.35)	2.93 (0.57)
Agreeableness	3.25 (0.62)	3.84 (0.56)	4.07 (0.34)	3.24 (0.22)	3.01 (0.36)	3.22 (0.83)
Externalizing problems	1.47 (0.27)	1.08 (0.10)	1.12 (0.10)	1.31 (0.19)	1.21 (0.08)	1.22 (0.16)

Before the intervention, there were significant differences in data between the experimental group and the control group, so the pre-test data were used as covariates. The results of the repeated measurement ANCOVAs are given in Table 6.

**TABLE 6:** Analysis of covariance analysis before and after intervention.

Dimension	Source	*df*	*MS*	*F*	η^2^*_p_*
Conscientiousness	Group	1	11.884	40.161***	0.378
	Time	1	0.093	0.238	0.004
	Time × Group	1	0.062	0.159	0.002
Agreeableness	Group	1	24.278	75.758***	0.534
	Time	1	0.519	1.714	0.025
	Time × Group	1	0.005	0.942	0.000
Externalizing problems	Group	1	0.577	44.596***	0.403
	Time	1	0.031	2.872	0.042
	Time × Group	1	0.001	0.072	0.001

PS: ***p < 0.001.

In the conscientiousness, agreeableness, and externalizing problem behavior, the main effect of Time and the interaction between Time and Group were not significant. The main effect of Time indicated that there was no significant difference in the scores between T2 and T3. The main effect of Group was significant. The main effect of Group indicated that experimental groups individuals reported higher scores on conscientiousness and agreeableness, but lower scores on externalizing problems than control group individuals in general.

The results revealed that the intervention not only improved the level of conscientiousness and agreeableness, but also reduced the level of externalization problems. The effects of the intervention were maintained at the 4-month follow-up.

## Discussion

The intervention goals play a guiding role in the experimental process (Tammemagi et al., 2013). Therefore, the training or intervention is more targeted. Previous intervention studies have found that appropriate intervention goals can have a good effect (Dunford, 2011; Bruhn et al., 2016; Vroland-Nordstrand et al., 2017; Yang et al., 2019). The reason for this study’s good intervention effect is that the intervention goals were aimed at improving the undercontrollers’ externalizing problem behaviors. The results of this study are consistent with previous studies, as we found that undercontrolled primary school students exhibit various externalizing problems that are associated with the conscientiousness and agreeableness personality dimensions (Jones et al., 2011; Frick and Ray, 2015).

We targeted intervention goals to the externalizing problems that undercontrolled primary school students experience in their actual lives. Through qualitative research, we collected examples of specific behaviors that are thought to be associated with seven facets under the two personality dimensions. For example, in terms of self-control, “Learning and doing things often go halfway and it is difficult to stick to one thing” and “Contradictions with classmates and inability to control impulses” are behaviors exhibited by undercontrolled personality types that have been found in previous research (Bohane et al., 2017). For these behaviors, we set the intervention goals of learning consciously, controlling impulsive emotions, and continuing to work hard when they encounter learning difficulties. In terms of concentration and responsibility, through observations and interviews with teachers, we determined the following problem behaviors that students displayed: “Inattention during class,” “Learning to do things sloppy,” and “Not actively participating in organized activities and not being concerned about class matters,” the intervention goals put forward by parents and teachers was “Listen carefully, stay focused, and learn and work undisturbed; Do not shirk responsibility, and have a sense of responsibility for the class.”

The results showed that the intervention realized the goal of reducing externalizing problems by changing the personality dimensions of agreeableness and conscientiousness. There are two main reasons for this result. One is to select the systematic experiential mental health activities as the best carrier. These activities can fully attract children’s interest and attention, so that children with problem behaviors can gradually internalize positive behavioral performances. The second is to compile the system experiential mental health activity program. The activity program is compiled under the guidance of intervention goals. The intervention goals were designed around the specific seven facets under the two personality dimensions that have a significant relationship with externalizing problems. Our findings support the idea that targeting intervention to these personality dimensions can effectively improve the externalizing problem behavior of undercontrolled children. For example, to target the self-control facet in the conscientiousness dimension, we designed the “Self-control skills of martial arts” activity. In this activity, we first presented a conflict situation, and then, the students role-played the situation. After this experiential activity, students discussed the situations in which impulsive behavior tended to occur and the consequences. Finally, we asked the students to summarize their strategies for controlling their impulsive behaviors, which they compiled as “martial arts secrets,” and to then stick them on their desk to remind themselves to reduce the occurrence of impulsive behaviors.

Four months after the intervention, we tested the personality and externalizing problem behaviors of undercontrolled primary school students again. The results showed that in the conscientiousness, agreeableness, and externalizing problem behavior, the main effect of Time was not significant. It can be seen that the effects of the intervention were maintained at the 4-month follow-up. In the process of interviewing the teachers, we found that the undercontrolled children had little or no more impulsive and aggressive behaviors through intervention. When there are conflicts in the class, the undercontrolled children also actively mediate the conflicts and advise him or her to think from the other’s perspective. The reason is that when the undercontrolled children perform well, teacher’s encouragement and praise will play an important role in the efficacy of the intervention. It may also be that children adhere to internalized moral values, which might make them show prosocial behaviors such as generosity and helping others after participating in the intervention activities (Benish-Weisman et al., 2019). In addition, children gain positive emotional experience through intervention activities, which will also increase the frequency of spontaneous prosocial behavior.

### Limitations

There are two main limitations in this study. One of the limitation is the Personality Inventory were responded for teachers, while the CBCL were responded for parents. Both scales were used to assess the effects of the intervention on improve externalizing problems. Teacher’s assessment of children was based on their performance in school. Parent’s assessment of children was based on their performance at home. However, children may exhibit diametrically opposite behaviors or different degrees of similar behavior in two different environments. Parents and teachers have different assessment because of the different perspectives of knowing children. This may have a certain impact on the research results. Another limitation is that this study used only questionnaires to measure the effect of intervention, and although they were multi-source, the results could have been supported by the application of other data collection techniques such as continuous observational records from parents or teachers and real-time data collection through apps.

## Conclusion

Results showed that the system experiential mental health activity can reduce externalizing problems of undercontrolled children by enhancing the personality traits of conscientiousness and agreeableness. Furthermore, 4 months after the intervention, the system experiential mental health activity demonstrated a sustained promotion effect.

## Data Availability Statement

The datasets for this article are not publicly available because the topic is not finished. Requests to access the datasets should be directed to the corresponding author.

## Ethics Statement

The studies involving human participants were reviewed and approved by the Committee of Liaoning Normal University. Written informed consent to participate in this study was provided by the participants’ legal guardian/next of kin.

## Author Contributions

YY, LY, and YS designed the experiment, prepared the materials, and performed the experiment. YY, LY, CJ, and YZ analyzed the data and wrote the manuscript.

## Conflict of Interest

The authors declare that the research was conducted in the absence of any commercial or financial relationships that could be construed as a potential conflict of interest.
