# Supplementary material for: Intervention on Externalizing Problems of Undercontrolled Personality Types in Primary School Students
Source: Front Psychol. 2020 Jun 23;11:1233. doi: 10.3389/fpsyg.2020.01233 (PMC7344270; doi:10.3389/fpsyg.2020.01233)
Supplement: Supplementary file 1 [file Data_Sheet_1.PDF]

Supplementary Material

Supplementary Material

1. Supplementary Figures

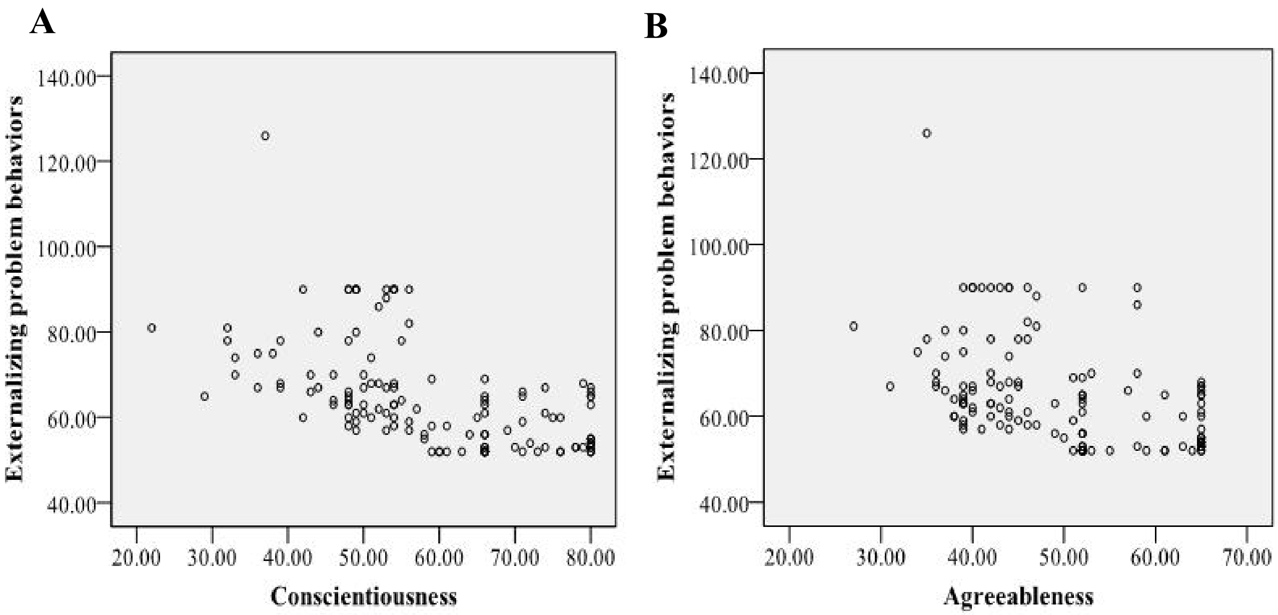

Figure S1. Correlation between personality dimensions and externalizing problem behaviors (EPB), (A) Conscientiousness and EPB, (B) Agreeableness and EPB.

2. The activities of the program

2.1 Concentration and Responsibility

2.1.1 Activity name: Focus my attention!

(1) Purpose of the activity:

✧ Make students realize the importance of concentration.

✧ Enioy the joy from concentration.

✧ Master the method of concentration.

(2) The record of materials

✓ Courseware; "Kitten Fishing" video

✓ Children's favorite movies

(3) Descriptions of the activity

Students are invited to watch the video of kitten fishing.

Teacher: What are the reasons why the kitten did not catch the fish?

Teacher: Why did the kitten catch a fish later? Today we are going to study "Focus on my attention" together.

Game 1:Reverse action

Rules of the game: Students need to make the opposite action according to the instruction. For example, if the teacher says to turn left, the student will turn right. However, if he does not turn right quickly, he will not be able to continue playing the game. The last person to play the game is the winner.

Sharing: Let the winner talk about how he feels during the game activity and how can he did it?

What did everyone learn from this game?

Summary: Summarize according to the students' answers. Focusing attention can help us achieve the joy of success.

Game 2: Arithmetic competition

Rules of the game: All students calculate 25 arithmetic problems at the same time and complete it within five minutes, when you finished, please raise your right hand. In the process of students doing math tasks, teachers play movies that students like.

Sharing: The teacher asked everyone to discuss why some students do it fast, while others are slow.

Summary: In this game, the focused students are not only fast but also accurate. Students who did not complete the task are distracted by the movie. Therefore, when you are on a mission, control yourself to prevent interference from other things. Do you have a way to eliminate interference? How do you rule out interference? In order to be able to concentrate, let us summarize the methods of eliminating interference.

(4) Summary of the activity

Share: The activity is coming to an end, what are your gains and feelings?

Summary: Concentrated attention can enable us to effectively complete the task. Concentrated attention often makes us experience the joy of success. When the interference comes, don't watch, listen. When we focus on one thing, we can get praise from the teacher, admire from the classmates,

and more importantly, we can learn knowledge. Students who are unable to concentrate don’t worry, because concentration is not born, it can be slowly improved through hard work.

2.1.2 Activity name: Have a sense of responsibility.

(1) Purpose of the activity:

✧ Dare to admit mistakes, do not shirk responsibility

✧ Learn to forgive others for their mistakes.

✧ Be a responsible person.

(2) The record of materials

✓ Courseware; A place with enough activity space.

(3) Descriptions of the activity

Courseware shows three scenarios: a. Student A saw trash on the ground, and he said, "That wasn’t lost by me, it has nothing to do with me." b. Student B saw the water in the bathroom kept flowing, he said, "It’s not me who opened it." c. When the tools in the classroom were messed up, student C said: "It’s not that I messed up, I won’t sort them out."

Teacher: What kind of behavior is this? (Lack of responsibility)

Teacher: What is the sense of responsibility? Let’s talk about our understanding of responsibility.

Game 1: You say, I do

Rules of the game: Students need to react quickly after hearing the teacher’s instructions: raise the left hand when calling for one; raise the right hand when calling for two; turn left when calling for three; turn right when calling for four. Students who make mistakes should stand on the podium and apologize to everyone. Terminate the game according to the actual situation.

Sharing: ① When you make a mistake, how do you feel and what do you think? ② How do you feel when admit mistakes in front of everyone?

Summary: Everyone may make mistakes in the game. It’s like every one of us will make mistakes in life. After making mistakes, daring to admit mistakes is a kind of courage to take responsibility. The teacher wants everyone to be a person who dares to admit mistakes

Game 2: Scrape nose

Rules of the game:a. Divide the students into different groups, 6 students in each group. b. Two groups stand in two rows each time and stand face to face. c. Everyone has an opportunity to play the

"scissors, stones, cloth" game on behalf of your group with each other in order. d. The winner can scrape the loser's nose. The other losers need to complete 5 deep jumps as punishment.

Sharing:

① What's your responsibilities when you are preparing to guess a punch with the other party on behalf your group?

② How do you feel when you lose?

③ How do you feel when your companion fails? What do you think of the mistakes of others?

(4) Summary of the activity

There will always be wins and losses in the game. Everyone may make mistakes. Everyone is responsible their own actions. However, this game is a match between teams. As a team, everyone should be responsible for the team. One person lost and the group accepted the punishment together. The losing team members were in a painful mood. If the team members complain or blame him, he will feel even more uncomfortable. If the team members encourage each other, each team member who represents his team will feel the warmth of the team and work harder for the team to win.

2.1.3 Activity name: Attention training

(1) Purpose of the activity:

✧ Recognize the importance of concentration.

✧ Master how to eliminate interference for concentration.

(2) The record of materials

✓ Table tennis, Ping pong paddle

✓ Basin, Green beans, Paper cup, Chopsticks

(3) Descriptions of the activity

Game 1: The ultimate challenge of dribbling

Rules of the game:

a. The students participating in the dribbling of the table tennis rackets are the interference group, and the other students are the anti-interference group.

b. Randomly select the students as the interference group to stand in a row. The interference task is dribbling with a table tennis racket. The game requires students not to touch the ball with their hands

during the dribbling process. Students need to ensure that table tennis cannot fall on the ground during dribbling.

c. The sitting classmates play the anti-interference group. Their task is to do 20 arithmetic problems.

d. Ask the teacher's little assistant to write down the names of the students who are disturbed in the anti-jamming team and the number of times that they are disturbed. The students who are named and have more interference after the game ends will be punished by love. After the game started, the teacher wrote down the names of the students who were disturbed and the number of times they were disturbed. Students who are disturbed more often would be punished.

Sharing: Which student seldom drops the table tennis ball in the process of dribbling? Which students are disturbed more often? The students in the interference group and the interfered group are asked to share their feelings?

Summary: During dribbling, students need to stay focused so that they can complete the task. The students will definitely have a lot of mistakes if they don't focus on the task. As long as we do our homework seriously, we may definitely do well. However, if we do it while playing, mistakes will easily occur.

Game 2: Digging for treasure

Game rules: The student with the most "treasure" clipped with chopsticks will win in 1 minute. Children's familiar music sounded after the game started.

Sharing: How many "treasures" did you find? The students exchange experiences in the group.

Summary: Students who dig more treasures do not talk with other students, they are all focusing on their own affairs.

(4) Summary of the activity

Sharing: What kind of things do students need to focus on? How do we concentrate on doing things?

Summary: We must believe that we can concentrate on the class. We need to understand what we want to learn in advance. We need to follow the teacher's eyes in the classroom. We need to clarify the requirements of the task. In order to eliminate interference, we should control our curiosity. Attention is an important guarantee for us to learn and do homework.

2.1.4 Activity name: Happy race.

(1) Purpose of the activity:

- ✧ Responsible for the class.

✧ Give your own strength for the class to get honor.

(2) The record of materials

✓ 20 volleyballs, playground

(3) Descriptions of the activity

Step 1: Introduce the basic rules of the activity and the duties of the referee.

Step 2: Three referees are selected in the class.

Step 3: During the activity, the teacher observes the degree of student's participation and their behavior performance.

Rules of the game:

① The whole class is divided into two teams. Each team stands in series. Two people from each team form a group. Each time two groups come from the two teams to play the game. Each group needs to quickly run to the end where the volleyball is placed and get a volleyball. Two people in each group need to squeeze the volleyball with their backs while transporting the volleyball to the starting point. After the members of the previous group complete the task, the members of the next group quickly start the game.

② The other team members proceeded in sequence until all volleyballs were delivered.

③ The team with the highest number of volleyballs is praised as the speed team.

Sharing: How do you feel during the game ? What are the ways for the speed team to win? Please share with everyone. How does the referee feel? Please share with everyone.

(4) Summary of the activity

The duty of the referee is to supervise the students, ensure the smooth completion of the activity, and judge the performance of the players fairly. If there is no referee, the game will not proceed smoothly. Due to the efforts of members and the efforts of off-site staff, a team can achieve the final success. In order for the class to be honored, we should contribute our own strength. Because for the class, we need to be responsible.

2.2 Self-Control

2.2.1 Activity name: Obey the rules.

(1) Purpose of the activity:

- ✧ Establish a sense of obeying the rules and form the consciousness of consciously observing the rules and maintaining the rules.
- ✧ Understand the negative consequences of non-compliance.

(2) The record of materials

- ✓ Courseware
- ✓ Video of "Pedestrians Crossing the Road and Running at the Red Light"

(3) Descriptions of the activity

Game 1:The game of guessing word

Sharing: Which team first guessed it? Are you satisfied with the result of this game? What are the reasons for unfair, dissatisfied, chaotic scenes, and mutual interference between teams? Do you have any questions about this game?

Summary: This game lacks rules. The rules are the requirements that everyone must obey. Without rules, the game will not proceed smoothly. Now the teacher shows the rules of the game, let's play again!

Rules of the game:

① The teacher needs to say a word to the first member of each group.

② The first member can only describe words to other members through body language.

③ Supervisor: The teacher supervised the foul performance during the game. The game is limited to 4 minutes.

Sharing: The winning team shares the secret of winning. Which teams have fouled?

Game 2: Let's watch a video.

Sharing: After watching this video, what do you want to say? How do you feel? What are the effects or consequences of illegal traffic rules? What rules do we need to follow in school? What rules do we need to observe in society? What rules do we need to follow at home?

(4) Summary of the activity

Summary: Everyone knows a lot of rules! The teacher hopes that students will abide by the rules consciously at school, at home and in society.

2.2.2 Activity name: Perseverance keeps me progress.

(1) Purpose of the activity:

- ✧ Understand the importance of perseverance.

✧ Master the methods to enhance perseverance.

(2) The record of materials

✓ Courseware

(3) Descriptions of the activity

Game 1:Master, I can’t hold on!

Rules of the game:

a. Everyone’s arms are raised upwards in a "V" shape.

b. Raise one foot at the same time and stand on one foot.

c. Hold on for 3 minutes.

d. If you can′t hold on, you will shout "Master, I can’t hold on!"

Sharing and Summary: The teacher found that some students had poor perseverance. Do you want to strengthen your perseverance? How do we cultivate tenacious perseverance? I think some students already have some ideas. The teacher first tells everyone an inspiring story.

Game 2: Yamada’s story

Sharing:

a. How did Yamada Honichi win the marathon world long-distance running championship?

b. Does he set big goals or set small goals first? why?

c. If the goal is set at the end of more than 40 kilometers, the results will be different. why?

d. What have you learned from this story?

Summary: If you want to achieve the ultimate goal, you will decompose the goal into multiple easy-to-reach goals. Once you achieve a small goal, you will feel the joy of success. This feeling will drive you to achieve your next goal. You will eventually achieve your goals.

(4) Summary of the activity

Sharing: Let us share the harvest of the activity.

Summary: We have understood the importance of perseverance. There are many ways to improve perseverance. For example, set goals reasonably, cultivate interest, set benchmarks, etc. We hope that our students can master and apply them to our study and life.

2.2.3 Activity name: I want to control myself!

(1) Purpose of the activity:

✧ Cultivate students' persistence and self-control ability

✧ Master the method of controlling impulse.

(2) The record of materials

✓ Courseware

(3) Descriptions of the activity

Game 1:The game of "Blink"

Rules of the game: The whole class is divided into two groups to compete. Each group is assigned a member to play the "blink" game. The student who blinks first will fail. Other members can interfere with the opponent in order to defeat the opponent.

Sharing: How did you overcome the interference from others?

Summary: Impulse is the gunpowder in the bomb; Impulse is a regretful medicine. Impulsive emotions prevent us from focusing on everything. It will also bring a lot of trouble to our study and life. Therefore, impulsive emotions need to be regulated and controlled, which is very important for our primary school students.

Game 2: The secret of Martial Arts

Step 1: We should stop all behaviors and take deep breaths.

Step 2: We need to feel the physical and psychological response.

Step 3: What measure can we do? Why do you want to leave?

Step 4: Make the best choice..

Step 5: Music training. Listen to music for muscle relaxation training.

(4) Summary of the activity

The teacher asked the students to use martial arts secrets for role-playing. If everyone needs self-control, martial arts secrets will be a good choice. It enables you to control impulsive emotions and make sensible actions.

2.2.4 Activity name: Learn to wait

(1) Purpose of the activity:

✧ Understand the meaning and importance of delayed gratification.

✧ Master the methods to improve delay satisfaction.

(2) The record of materials

✓ The video of "Stanford Marshmallow Experiment"

(3) Descriptions of the activity

Game 1: Bet

Rules of the game:

The teacher needs to play a bet with the quiet classmates. Because quiet classmates do not talk indiscriminately during class, I need to reward them. If they need a reward now, they will get 1 marshmallow. If they can persist for 15 minutes without talking, they will get 2 marshmallows. If they talk indiscriminately for another 15 minutes, they will have no marshmallows to eat. Who wants to play this bet with the teacher?

Sharing: Who eats the most marshmallows? What have you learned from this bet?

Let us watch a famous experiment.

Game 2: The video of Marshmallow experiment

An aunt brought the child into a classroom and gave the child a marshmallow. The aunt told the children: if you eat right away, you will only eat one marshmallow. If you wait 20 minutes before eating this marshmallow, you will get two marshmallows. Let's take a look at the behavior of these children. ——Watch the video

Sharing:

a. What are the reactions of the children in the video?

Teacher: Some children can't wait any longer and eat the sugar right away. In order to resist the temptation of cotton candy, some children have done a lot of behaviors. For example, some children close their eyes, some children sing, some children talk to themselves, and some kick the table.

Summary: This kind of behavior that does not need to be satisfied immediately but the delay time is called delayed satisfaction.

b. What kind of children have low delayed satisfaction? What kind of children have high delayed satisfaction?

c. Does delay satisfaction matter to us?

The researchers visited these children again after 12 years. The study found that children who can wait longer for more marshmallows are more likely to succeed than those who are impatient. The assessment results of academic ability show that they are 20 points higher in math and language than

students with low delay ability. Children with delayed gratification show strong social competitiveness and can cope with the setbacks in life.

(4) Summary of the activity

Sharing: What do children with delayed gratification do while waiting?

Summary: a. Attention shift: children shift their attention from temptation to other things. b. Sour grape psychology: children believe that temptations are not as good as expected.

2.3 Planning and Orderliness

2.3.1 Activity name: Plan to lead learning

(1) Purpose of the activity:

✧ Cultivate students to develop the habit of making plans.

✧ Cultivate students' ability to arrange time.

(2) The record of materials

✓ Blank paper

(3) Descriptions of the activity

Game 1: Mutual understanding

Rules of the game: Each student introduces himself first and then introduces others. The content of the introduction is: My name, strengths, weaknesses. Everyone needs to introduce a familiar classmate in the same way.

Summary: I believe that every student will have a deeper understanding of themselves after listening to others' evaluation of themselves. For example, you have not found that you have beaten, sloppy and other shortcomings. You have not found that you have such good advantages. You need to say thank you for the classmates who helped you understand yourself. Each of us needs to recognize our own shortcomings.

Game 2: Draw and Write

Rules of the game: The teacher sent three blank sheets to each student. The three blank sheets are numbered. Students need to draw freely on the first white paper. Students need to draw a penguin on the second white paper. Students need to draw a little penguin with a yellow mouth, black body and white belly on the third piece of white paper.

Sharing: Which painting do students like best? Why?

(4) Summary of the activity

Learning without a goal, with a goal and with a clear goal is to produce different learning effects. Having clear goals allows us to learn efficiently. Setting goals is a common way to embody planning. We should achieve our goals step by step according to our learning plan. Through mutual supervision between students and reminders from parents, we can successfully complete the fourth grade learning tasks.

2.3.2 Activity name: How to spend weekends.

(1) Purpose of the activity:

✧ Guide students to plan a meaningful weekend.

✧ Learn how to make a schedule and arrange weekend activities.

(2) The record of materials

✓ A schedule

✓ Courseware

(3) Descriptions of the activity

Game 1: Debate Competition

The topic of our debate is “How to spend Saturday and Sunday?"

A: We should rest and play on Saturday and Sunday.

B: We should continue to study on Saturday and Sunday.

There are different opinions on how to arrange these two days. Some agree that on weekends we should learn to rest and learn to play, while others agree to continue to study on Saturdays and Sundays. Which arrangement is reasonable? Now the class is divided into two parties (A and B). Students who support Party A, please left hand and stand together. Students who support Party B, please right hand and stand together. Each member needs to state the supporting views and give reasons. Any student who has reason and evidence can get a five-pointed star.

Summary: How to spend weekends is very important to improve the quality of life of each of our students. We have to rest and recharge ourselves in these two days. We should combine work and rest to live a fulfilling life.

Game 2: My Weekend Plan

a. Teacher asked students to design a schedule for this week’s weekend.

b. The students learn from each other's schedules. Students are asked to post the schedule on the blackboard for everyone to learn.

(4) Summary of the activity

The relaxed and happy weekends provide each of our classmates with learning opportunities and an arena for activities. Let's start a wonderful and meaningful weekend this week.

2.3.3 Activity name: My time is up to me.

(1) Purpose of the activity:

- ✧ Understand the importance of arranging a reasonable time.
- ✧ Guide students to plan a reasonable time.
- ✧ Master the method of managing time.

(2) The record of materials

- ✓ Courseware, Blank paper

(3) Descriptions of the activity

Game 1: One minute of mystery.

Rules of the game: Students need to memorize 10 words in one minute. Students need to read a poem in one minute. Students need to do 5 math problems in one minute. Students need to do push-ups for 1 minute. Students need to close their eyes for 1 minute.

Sharing: After the game, what do you want to say?

Summary: When we do nothing, time passes slowly, but when we concentrate on doing one thing, time seems to pass quickly.One minute may seem short, but we may do many things. 70 words per minute, 45 push-ups per minute, 200 to 400 meters per minute, you can skip the rope 100 to 200 times a minute, and one minute will be fleeting! If time is used reasonably, many things wil be done in one minute.

Game 2: Four-quadrant rule of time management.

One day, a classmate was very anxious. Because there were many things to do in a day. He had a piano exam at 2pm and had not yet intensively reviewed. He made an appointment with his classmates today to play basketball. He was going to take a taekwondo class at 10 am. He needed to watch a TV series for a while.

Sharing: If you were this classmate, what would you do?

Summary: This classmate can use the four-quadrant rule. It divides things into two dimensions of importance and urgency. Things can be divided into four quadrants according to their importance and urgency. The four quadrants are important-urgent, important-not urgent, unimportant-urgent, unimportant-not urgent.

a. The first quadrant is important and urgent

It refers to urgent and important things. This kind of thing has the urgency of time and the significance of its impact. It cannot be avoided or delayed. It must be dealt with first and resolved first.

b. The second quadrant is important but not urgent

It is not urgent in time, but it has a major impact. Many important tasks in life and work need to be prepared before the event.

c. The third quadrant is urgent but not important

It refers to urgent but unimportant things. Unimportant events often take up a lot of precious time because they are urgent.

d. The fourth quadrant is neither urgent nor important

It refers to things that are not urgent or important. We will waste a lot of precious time and energy doing these things.

(4) Summary of the activity

Sharing: How would you arrange things in the story according to the four-quadrant rule?

Summary: Afternoon piano lessons are important and urgent. So this should be done first. Playing basketball is important but not urgent. So playing basketball is about to finish the exam in the afternoon. Taekwondo class is urgent but not important. He can watch TV after playing basketball. Time management is an important strategy for self-regulated learning. Effective time management can promote learning and enhance self-efficacy. Ineffective time management will weaken confidence and reduce learning efficiency.

2.3.4 Activity name: Methodically.

(1) Purpose of the activity:

- ✧ Learn to develop a reasonable schedule.
- ✧ Experience the sense of accomplishment brought by orderly completion of tasks.

(2) The record of materials

- ✓ Courseware

(3) Descriptions of the activity

Game 1: Observation Island

The courseware presents two scenes for comparison. In the first scene, the boy is writing his homework with the table messy, looking at a pile of books in a daze. He doesn't know what to do. In the second scene, the girl records the homework assigned by the teacher one by one in the school. She planned the homework she needed to do. After returning home, she did her tasks in an orderly manner.

Teachers: which student do you prefer? why?

Students are asked to express their ideas and opinions.

Summary: When we face many tasks, careful planning in advance can save time and improve efficiency. Today let's learn how to reasonably formulate a suitable study plan.

Game 2: Exclusive customized schedule

The plan should be feasible. The previous plan lacks flexibility and is difficult to implement. Let's analyze others' schedules together. What is missing in analyzing your original plan?

My starting point

Subject   Original grade   Self-evaluation   Reason

My goal

My dream is to be a ______________________

I plan to reach _______ points in the final exam of this semester.

Please make a review plan before the exam:

My review method before the exam

① Review of basic knowledge.

② Master the solution of various math problems.

③ Remember knowledge through understanding.

Combine your test experience and our study, please write your test skills

What do I prepare before the exam? __________________________________

What is my review method? __________________________________

How do I overcome test anxiety?_____________________________________

Summary: In the review stage, students need to find a suitable study plan for themselves. A suitable study plan will have a good learning effect.Everyone needs to write down their overall goals and stage goals. Everyone needs to develop a learning plan for their goals. Specific practices need to be written on the schedule. The teacher will select the best plan.

(4) Summary of the activity

We have a lot of homework and tasks to complete. Making a clear, orderly, and operable learning plan for yourself can improve our learning efficiency. Students can prepare a plan for themselves before the semester begins. This schedule can be detailed to monthly or weekly. You can also make a specific small plan for daily study.

2.4 Aggression and Resistance

2.4.1 Activity name: Methods other than attack.

(1) Purpose of the activity:

✧ Be able to reflect on your own actions and tolerate others.

✧ Guide students to handle conflicts among classmates correctly.

(2) The record of materials

✓ Courseware

(3) Descriptions of the activity

Game 1: You catch me and I escape

Rules of the game: Everyone raised your left hands at shoulder height and put your palms down. Raise the index finger of your right hand upwards and press the palm of the classmate next to you. The teacher tells the story of "Crow and Turtle". When hearing "Crow", all students should use your left hand to grab the right index finger of the next classmate, while avoiding your own right index finger being caught. Who responds quickly and accurately?

Game 2: Brainstorming

Case 1: Hearing the bell after class, Xiao Fan can’t wait to rush out of the classroom. When passing by Xiaohong’s seat, Xiaohong’s stationery fell to the ground instantly. Xiaohong is very angry. If you are Xiaohong, what do you want to do next?

Note: The teacher writes down all the ideas that everyone thinks about. Then let students discuss what kind of consequences (pros and cons) this kind of practice will bring. Students analyze and evaluate and find the best solution.

Case 2: In the self-study class, Xiao Wang Jinjin reads books with interest. Suddenly, Xiao Tao at the same table grabbed the textbook and fell hard. He lost his temper and accidentally hit Xiao Wang. The two started a dispute. Xiao Tao was already angry, at this time, it was even more fueled by the fire, and they did not give in to each other. Everyone brainstormed, and from the perspective of them, talk about what Xiao Wang and Xiao Tao felt and thought at the time? (Role play can be added) The two are about to fight, everyone find a way for them to solve it.

Note: The teacher records all the methods and analyzes the consequences of each method to find a suitable solution.

Summary: Some people feel that when facing problems, we need to think hard about solutions and put them into action. Some students think that we can ask parents and teachers to learn how to solve problems. Very few people will talk badly and fight.

Discussion topic: How to prevent your own aggressive behavior against others?

① Speak it out: If you are very annoyed by someone and want to “clean up” him, before doing so, be sure to tell the parents, teachers or seniors. They will come up with a reasonable solution to your anger.

② Think from the perspective of the other party. If you were fooled, abused or even beaten, how would you feel? If your actions bring long-term fear and pain to others, will you be patient?

③ Ask yourself: Is it worth reflecting on it? What you have to do may bring you criticism, punishment and even legal punishment, which will become a stain on your life. The cause may be a small thing, or just your own impulse. Is it worth it?

④What is the result? Is it helpful to solve the problem?

(4) Summary of the activity

We must cherish friendship and pay attention to courtesy. Taking the initiative to reconcile with people is not only a loss of face, but also a sign of generosity and maturity. A "sorry" is enough to see sincerity; a "please forgive" will not lose your identity; a "thank you letter" to express your sincerity; a "third person", build a communication bridge; dilution is unpleasant; a "sincere heart" turns stubborn stone into gold.

2.4.2 Activity name: Communication strongman.

(1) Purpose of the activity:

- ✧ Students can correctly understand the care and education of teachers and parents for themselves.
- ✧ Students exchange their own methods for resolving conflicts, discuss and share their successful experience in resolving conflicts.
- ✧ Students understand the psychology of rebellion and the harm caused by the psychology of rebellion, and master the countermeasures to overcome the psychology of rebellion.

(2) The record of materials

- ✓ Courseware

(3) Descriptions of the activity

Game 1: Build a friendship home

Each group collectively builds its own friendship home, then select a homeowner.The host organizes a group discussion. Discussion content includes:

a. Friend’s name.

b. The shape, layout and furnishings of the Friendship House.

c. The friendship tenet or slogan advocated by the Friendship Home.

Each homeowner draws on the blackboard the shape, layout, furnishings of the Friendship Home constructed by the group and explains the name, friendship purpose, or slogan of the home. Please each team member to add some strokes to the blackboard.

Sharing: Ask each group to send representative to share the discussion results of the group with the whole class.

What qualities do you think members of your friendship family should have?

If there are conflicts among members? What should I do if a conflict occurs?

How do you think the friendship members can live in harmony?

Note: Remind students that the home can be a group of teachers and students.

Summary: The friendly family that everyone builds has a class like everyone, as well as a warm family composed of parents and grandparents. There will be various contradictions between the family members. Various contradictions are inevitable, because each of us has our own unique ideas and thinking. When the ideas are not the same, it will produce contradictions; but we should learn to

think empathically. Both parents and teachers care and educate us. When conflicts and conflicts arise, we communicate calmly on the basis of tolerance. Even if we are reasonable, we should not be unreasonable. If we are reasonable, we will not give in. The result will inevitably make it difficult for the other party to step down and intensify conflicts. For this reason, o resolve contradictions and conflicts should adhere to the principle of "reasonable and polite" and learn to give in. If you are unreasonable, you should take the initiative to admit your mistakes and honestly apologize. Don't make it unreasonable for the sake of the face. Let teachers and parents forgive your youth and ignorance and some inappropriate behavior and we should have full trust in teachers and parents. In fact,there is no doubt that teachers and parents want their students and children to be good.

Game 2: I am him, but he is not me

Case: Sun Wei, a 10 years old boy, is a fourth grade primary school student. He often clashes with parents and teachers. At school, his behavior of rebellion is very sharp. Whenever the teacher criticizes him, his eyes are directly on the teacher, a look of dissatisfaction, and he even talks back to the teacher. In class, he deliberately spoke and made small movements, and he could not complete his homework after class.

What attitude does Sun Wei show to parents and teachers?

What is reverse psychology? What are the specific performances?

What harms does rebellious psychology bring?

Do you have similar experiences in your study and life? Please give your example.

Summary: The conversations of these classmates show their disobedience and resistance to their parents.These conversations reflect the rebellious psychology of primary school students in real life. Guide students to analyze the harm caused by it from multiple angles. It leads to a loss of interest in learning, a loss of confidence in life, and it is extremely detrimental to physical and mental health, causing psychological deviation and hostility for ourself. It affects the parent-child relationship and severely leads to family division for family. For other aspects, it leads to tension between classmates, teachers and students, and relatives and friends, which affects your image and is not conducive to your own development and progress.

(4) Summary of the activity

Summary: We must understand the true intentions of parents and teachers. Learn to grasp yourself and be able to stabilize and restrain emotions. Actively communicate and effectively solve problems.

Cultivate divergent thinking and consider issues in many ways. Improve our cultural literacy and psychological adaptability. We have to learn to think differently and get out of the misunderstanding of "working against parents and teachers".

2.4.3 Activity name: To turn war into silk.

(1) Purpose of the activity:

✧ Understand aggressive behavior, realize that aggressive behavior is wrong, and experience the feeling of being attacked by others.

✧ Know the negative impact of attacks, and master the methods to prevent or reduce attacks.

(2) The record of materials

✓ Courseware

(3) Descriptions of the activity

Game 1: Look at the picture

Sharing: Watch pictures and talk about these behaviors of these children's performance.

Summary: Robbing toys, beating people, pushing others, throwing things, cursing, etc, we call these behaviors offensive behavior. Aggressive behavior refers to the behavior of intentionally harming others, damaging or robbing other characters. It manifests in three aspects: one is direct physical attack, such as twisting and twisting others, smashing with fingers, squeezing ,pushing and scratching others. The second is verbal attacks, including swearing, ridicule, nicknames, etc. The third is indirect attacks, including malicious creation of remote, instigation of beatings, social exclusion, etc.

Sharing: Are these actions correct or wrong?

Summary: Regardless of the cause and under any circumstances, offensive behavior is wrong and undesirable, it will cause irreparable consequences and harm. The teacher brought you a real campus attack. (An American 10-year-old girl school fights and dies). How do you feel?

Game 2: Case discussion

Yuhao is a fourth grade primary school boy. His brain is flexible and he was slightly naughty in the first and second grade, but he did not arouse special attention from teachers and classmates. Starting from the third grade, he gradually showed the characteristics of grumpy and fighting. When someone said a word to him or touched him accidentally, he would rush to the crown and hit him. He also has a particularly sensitive place, that is not allowe others to touch his head, even in a friendly or

pampering way, he will be angry. Because of his bad behavior, the students are very afraid of him, no one wants to ignore him, and play with him. Nowadays, it seems that Yuhao has become a habit of hitting people, even inexplicably pushing others, or suddenly picking up the pen at the same table in the middle of the class, and sometimes running to other classes to provoke trouble. Almost every day, students who were bullied by him came to the office to complain. When the teacher educates Yuhao, he will admit his mistakes, even crying weepingly to ensure that he will no longer hit people, but often only quiet for a day or two.

Sharing: Why does Yuhao have no friends? How did he do it? What are his attacks? What is the impact?

Summary: Yuhao’s performance is a typical offensive behavior. Attacking others will have a bad influence on other people’s psychology and body. For a long time, no friends are willing to be with him. Are there any classmates like Xiang Yuhao around you?

Game 3: I will be an actor-How to stay away from or correct my aggressive behavior.

cosplay:

Sha Sha and Jia Jia are drawing, and Jia Jia lacks a red crayon. When she sees that there is exactly one in the Sha Sha’s box, she reaches for it, and she says, "This is mine." Sha Sha was about to use it, and refused to give Jia Jia. Jia Jia was so angry that threw away all the things Sha Sha painted, pushed her to the ground, and kicked with his feet.

Sharing:

What is the behavior of Jiajia, what kind of behavior does it belong to, and what is the reason for the behavior?

How to prevent and stop the occurrence of aggressive behavior?

Summary: The behavior exhibited by Jiajia is offensive. The reason for the behavior is that she has no red crayon and there is no effective communication. It should make her realize that attacking others is wrong. Before borrowing something, you need to get the consent of others. Students should care for each other, unite and love each other, and if there are conflicts, they should be resolved through consultation and reasoning- A man of honour reasons things out, and does not resort to force. Bear the calm, step back and spread the sky. Be calm and deal with contradictions. Seek the help of a teacher. Tell the parents to let them help.

Each of us has our own ideas. When our ideas conflict with others, we must learn to discuss and attack others can't solve the problem.

Control anger emotions, but how to control anger emotions?

(4) Summary of the activity

Learn to understand and tolerate.

Learn to keep silent.

Distract attention.

Delay the reaction.

Learn some simple relaxation methods, such as Breathe deeply.

2.4.4 Activity name: Please help me.

(1) Purpose of the activity:

✧ Through self-assessment and other reviews, he realized his bad behavior of attacking others

✧ Learn to control his own attacks on others.

(2) The record of materials

✓ Courseware

(3) Descriptions of the activity

① Group of about 10 people, sitting in a circle. Everyone spends two minutes talking about their good behavior and bad behavior.

② Place two seats in the circle, face to face, one for the person who receives the feedback, and the other for the person who gives him the feedback. Find out the good behavior and bad behavior of the other party.

③First, one student sits in the seat receiving the feedback, and the other students take turns taking the seat giving him the feedback. Everyone's feedback to him should be true and specific, such as "you fight", "collide with the teacher", etc. No one else can express an opinion. The students who receive the feedback don't say anything, just listen. One student receives the feedback from all other students, then another student will take the seat and proceed as described above until all the students gave him feedback.

④The teacher leads the discussion.

Summary: Not only does everyone have good behavior, but also more advantages and progress.I will look for volunteers who want students to find good behavior and want to improve, and select the students to be intervened from the raised hands.

⑤The teacher tells you how to correct bad behaviors. Reverse the roles or find a blank piece of paper and write the word Ninja 10 times.

⑥ Assignment: Students in the intervention group write "I will change 12", list the 12 good behaviors that may appear recently, and they must be specific, true and feasible (estimate the specific and feasible progress that they will appear recently, which is positive self-prediction).The teacher and classmates keep a "secret red account" and secretly record the observed good behaviors and advantages of interfering with the students.

⑦ Read the red account: the classmates and teacher reports the good behaviors and progress of the interventions secretly recorded in the class to consolidate the observation effect and strengthen the good behavior and progress that occur.

⑧Say "I will change 12": Intervene students read the last assignment "I will change 12".

2.5 Sympathy and Altruism

2.5.1 Activity name: Friendship for the disabled.

(1) Purpose of the activity:

✧ If you encounter difficulties for the disabled, try your best to help and actively participate in disability assistance activities in schools and community organizations.

✧ Do not mock or mock the disabled.

(2) The record of materials

✓ Courseware

(3) Descriptions of the activity

Game 1: Stick the nose

Two students at the same table as a group, one plays the armless person whose hands and arms cannot move, and the other plays the blind person with the red scarf covered eyes.

After the game started, the two set off from the starting point and cooperat to stick the nose to the person without nose. The group that correctly attaches the nose to the non-nosed person will be added one point.

Sharing:

What is the reason for winning? Ask students to imagine what difficulties the blind person will encounter in their study and life?

In addition to the blind, who are the disabled? Tell one of them and what difficulties will they encounter?

As a normal person, how should we treat and help them?

Summary: In normal life, we do not feel such pain and inconvenience. First of all, we must give them care and attention in life. Try to help them solve their difficulties. Furthermore, we can give them encouragement and support in spirit, and relieve them of the pressure and pain in their minds.

Sharing: Why should we help people with disabilities?

Summary: People with disabilities will encounter many difficulties in life. They will have some special situations both physically and psychologically, so we need to care, sympathize and help them. Disabled people are very unfortunate, but they should enjoy the same treatment as normal people and enjoy dignity in personality. We must respect them. In our daily life, there are some people, although disabled, they continue to strengthen themselves, overcome many difficulties, and make a great contribution to society, which deserves respect.

Game 2: Come up with a situational question:

At the crossroads, I saw a blind man preparing to cross the street ...

In the carriage, I saw a crippled young man on crutches get in the car ...

On the way, I saw a handicapped aunt carrying something ...

On the campus, I saw a disabled classmate fall ...

Let students perform live.

On-site interview:

Visit a classmate who plays a normal person: Why do you help him?

Interview with a classmate who plays a disabled person: What did you think when he helped you?

Summary: Take the initiative to help disabled people solve difficulties. Take the initiative to give seats to disabled people when taking a car. Help disabled people to do what we can. Help students who fall.

(4) Summary of the activity

Helping people with disabilities is a manifestation of good social morality in society. Let's help people with disabilities in life, encourage and support them spiritually. Let us be a good boy of civilization.

2.5.2 Activity name: Caring for others.

(1) Purpose of the activity:

✧ Students know that helping others will bring happiness to themselves

✧ Through game activities, let students realize that helping others and others help me makes me happy.

(2) The record of materials

✓ Courseware

(3) Descriptions of the activity

Game 1:The blind and crutches

Rules of the game:

a. At the same table, two people form a group to play the blind and the crutche.

b. The classmate who plays the blind uses a red scarf to cover his eyes and rotate 3 times in place.

c. The crutches supported the blind man with one arm, and the other arms of the two naturally sagged.

d. The blind man bypassed safely with the help of crutches but without touch our obstacles. Failure to do this is a foul, and you have to start from the beginning.

e. Finally reach the end.

Matters needing attention: Each group at the same table listen to the instruction of the teacher and come to the starting point. The students who successfully complete the task return to your seats. During the entire game, all the students who are sitting are the supervisors like the teacher to supervise who fouls! In the second round, the roles are exchanged.

Sharing:

1) What do you think about blind people's psychology when they are guided as blind people? What if there is no help from crutches?

2) As a crutch, what do you think when you are dependent?

Summary: I feel happy when I am helped, it is difficult to do things without the help of crutches. People with disabilities should be cared for more often. Leading the blind to the end makes me happy.

Through the sharing of the students, the teacher also deeply fells the deep experience of the students. When we are helped, we are happy. When we help others, we are also happy.

Game 2: "You said, I said"

In actual life, how can we be a person who will care for and help others? Now let's walk into " You said I said ". Let's take a look at the three scenes presented in the PPT. Each group discusses one of the scenes. What would you do in such a scene?

The teacher shows three scenes separately and say:Now please discuss in groups.

(4) Summary of the activity

Through the sharing of students in various groups, the teacher feels that you are all very caring students. The teacher also hopes that everyone in daily life can help more people who need help.

2.5.3 Activity name: Gifts of roses.

(1) Purpose of the activity:

✧ Guide students to realize that helping others is a happy thing

✧ Guide students to learn to understand the worries and pains of others, and recognize the important role of care and sympathy

(2) The record of materials

✓ Courseware

(3) Descriptions of the activity

Game 1:Jigsaw puzzle

Rules of the game:

① There are 8 numbers on the blackboard, please select one number for each group.

② Each number represents a kind of jigsaw puzzle. There are 8 kinds of jigsaw puzzles of different difficulty. After selecting all, the teacher sends jigsaw puzzles to each group.

③Members of the completed team will receive 1 point, and then the members can sit on their seats to watch the battle, or they can turn into angels and fly to the unfinished group to help them complete the puzzle.

Sharing: Please the group that got these two medium difficulty pictures stand up. Who do you think you are most grateful for completing this puzzle? The group that got the hardest puzzle please stand up. Although you haven't finished, we all know that your puzzle is the hardest. You have been

working hard to let us all applaud and encourage you. I want to ask you although you have not completed it, who are you most grateful for? Now please stand up as an angel during the game. Why do you help them?

Summary: These angels did not help others for any rewards, but for helping others, let's give them the warmest applause.

Game 2: Psychological focus

Sharing: Have you ever helped others? How do you feel after helping others?

Summary: Gifts of roses, hand left lingering fragrance! Helping others doesn't have to be vigorous. Please support him when the classmate falls. If something at the same table drops, I'll help pick it up. I give the old man a seat. When others are studying, I will keep quiet. These small movements are helping others, and raising hands, why not do it?

(4) Summary of the activity

Summary: Our class is an excellent class collective. In this collective, we help each other and live in harmony, just like a big family, full of warmth and love! I hope that everyone of you will take the initiative to help everyone in need. Not only for behavioral help, but more importantly, to truly care for classmates from the heart and understand the difficulties of classmates. When meeting classmates who are sad, as a person around them, learn to comfort and care for them.

2.5.4 Activity name: Compare heart to heart

(1) Purpose of the activity:

✧ Learn to be considerate of others.

✧ Learn to pay attention to the unfortunate and poor people around you or in society, and cultivate compassion and love.

(2) The record of materials

✓ Courseware

(3) Descriptions of the activity

Game 1:They need our help.

Let's listen to a song about love "Dedication of Love". Let's think about what this song sings. Where and when did you hear this song? What do you think of this song?

Teacher: We can say that we live in happiness and joy. At this time, have you ever paid attention to some people around us who have a difficult and unfortunate life?

I have encountered ( )

I saw ( ) on TV

I saw ( ) in the newspaper

I heard from mom and dad that ( )

I found it online that ( )

Teacher: There are many people around us who are sick or have difficulties in life. They need our help. As long as everyone gives a little love, the world will become a beautiful world.

Game 2: Learn to help others.

Invite students to watch "What Should I Do"

Group communication

Invite students to watch "What Should I Do"

Everyone discusses in the group:

What difficulties did Yan Yan encounter? What was her mood at the time?

What should Yan Yan do at the moment? What can we do for her?

Teacher: When Yan Yan’s parents are seriously ill, they will be very physically and psychologically painful. At this time, we should offer them love. This love is not just material, but also spiritual encouragement and psychological comfort.

(4) Summary of the activity

We are a group of lucky people who can live in happiness, but we cannot ignore the people who live in hardship and misfortune. When someone encounters misfortune and has difficulties in life and study, don′t forget to extend our hands and we should think fort others and offer our love, because as long as everyone gives a love, the world will become more Beautiful.

2.6 Gregariousness and Courtesy

2.6.1 Activity name: Cooperation skill.

(1) Purpose of the activity:

✧ Students can be well integrated into the group and interact well with their peers.

✧ Students can effectively collaborate with others in activities to achieve goals together.

(2) The record of materials

✓ Balloons, cards,

- ✓ Straps, outdoor venues

(3) Descriptions of the activity

Game 1: Blowing balloons together

Ready to work:

Teachers should prepare cards according to the number of students, issue 6 cards to each group, write "hand" on two cards, "foot" on two cards, "mouth" on one card, and "butt" on one card. Each group has a balloon.

Rules of the game:

① All the students form a circle with the teacher as the center, and the 6 students next to each other form a group.

② Each group of students formed a circle and sat down.

③ Each group sends a student to collect cards and balloons. Each one in the group draws a card. The student who draws "mouth" can only use his mouth to blow the balloon, and the student who draws hand helps the mouth blow the balloon with his hand. Students who reach the butt must use their butt break the balloon, and those who reach the "foot" need to lift the students who reach the butt.

④The team with the fastest completion and the shortest time wins.

Share: How do you feel by playing this activity？ Please the members of winning group comment on your group members. If you are given a chance, how can you make your team win?

Game 2:Tacit partner-relay race

The first round of game:two-person three-legged game

1) The two people in the first row tie their adjacent legs together with straps and walk from the starting point to the ending point, and then back to the starting line. The previous group and the next group of students clap their hands, and the next group of students start walking again. The first group of students quickly release the bandage and tie the bandage to the third group of students.

2) During the advancement, the strap cannot fall off and the body cannot touch the ground, otherwise please return to redo.

3) The team with the fastest completion wins.

Note: The teacher gives you 1 minute to practice before starting. The first round of activities begins and students compete, while teacher does a good job of supervision. Students perform the first round

of exercises in the group. Teachers should guide students to actively participate in exercises and answer students' questions in a timely manner.

Sharing: What are your feelings and thoughts through this activity, what factors are driving your group to win, and why did you failed? If give you another chance, how can you make your team win? Some students' legs are hurt where they are tied. What causes pain? What are the consequences of inconsistent pace? Who was trampled on during the activity, how did you do it, and why?

Summary: Unite you and me.Put friendship first, match second and the pace is consistent. One person to command is the key to winning. Practice once or twice before the game to enhance the tacit understanding. During the activity, everyone tied their leg together with others and there were many times when they stepped on their feet, but everyone endured the pain and persisted, which was great. Although there were a lot of frictions and difficulties, as everyone tried again and again, and finally we achieved success. It is normal to cooperate with classmates to create frictions and conflicts, because tacit understanding is produced by continuous running-in. The key is how we treat it.

Second round of activity rules

a. Standing in line with the team just now, now six people in each team stand in a row.

b. Tie the legs of the two people together with a cloth strip and the second row is ready.

c. In the process of forward or backward, the cloth can't fall off and the body can't touch the ground, otherwise it will return to redo.

d. The team with the fastest completion wins.

Note: Students practice the second round of activities in the group. After the practice, the second round of activities begins and the students compete. Teacher does a good job of supervision. At the end of the activity, the teacher asks questions in turn, and students communicate and answer in the group.

(4) Summary of the activity

Games let us understand unity, communication, cooperation, tolerance and perseverance

2.6.2 Activity name: Take the initiative.

(1) Purpose of the activity:

- ✧ Learn to actively communicate and interact with your peers.
- ✧ Students can effectively collaborate with others in activities to achieve goals together.

(2) The record of materials

✓ Outdoor playground

(3) Descriptions of the activity

Game 1:Squirrel and big tree

Rules of the game:

1) All students form a circle with the teacher as the center, and count according to 1, 2, and 3 to form a group. 1 and 3 is a big tree, 2 is a squirrel. You play the same role. For example, the classmate who plays the big tree has always been the big tree. It can't become a squirrel in a while. If there are surplus students, they're angel, if not, the teacher is the angel.

2) Two big trees put their hands on each other's shoulders, and the squirrel squatted under the big tree (Teachers need to make good demonstrations);

3) The teacher has three passwords.a: The hunter is here. b:It is on fire. c: The earthquake. When I say the hunter is here, the big tree does not move, and the squirrel needs to be quickly transferred to the tree hole of the other big tree. When the fire is on, the squirrel does not move, the big tree needs to be quickly disassembled to form a new big tree with other big trees. When an earthquake strikes, both the squirrel and the big tree must change positions quickly, forming a new combination with other groups of squirrels and the big tree.

4) The angel's task is to play as a tree or squirrel after the teacher shouts the password.

Game 2: Change

① All students form a circle with the teacher as the center, and move clockwise or counterclockwise.

② I will give you different amounts of money according to your different characteristics such as one yuan for boys and five pentagons for girls, one yuan for wearing glasses and pentagons without glasses.

③ The teacher issues a password to give a total amount of money, and all the students quickly group according to the total amount of money given.

④ Students who fail to form the total amount of money correctly according to the password accept the punishment of love.

Sharing: Students who are angels in the pine and big tree games, please stand up, and those who placed orders in the change game, please raise your hands. Whether in the pine and big tree games or in the change game, there are some students who have been singled out.Who can tell the teacher what

causes a person to be singled out and others can express different opinions. How do you feel about this game?

(4) Summary of the activity

There is no initiative to find a team, and give others the opportunity to hold a group, and deny themselves; Some students take the initiative to find a team and combine with others.In daily life, do you miss some opportunities because you are not active? Say and share it with everyone.

2.6.3 Activity name: Cooperative minibus.

(1) Purpose of the activity:

✧ Through competitions, students can experience the joy of cooperation.

✧ Let students know how to cooperate with others.

(2) The record of materials

✓ Two Footballs

(3) Descriptions of the activity

Game 1:Cooperation Charm Ball

Hello classmates! Before the class, I want to ask everyone a question: What should you do when you see a teacher or a classmate greets you? These are two footballs. In ordinary times it is an ordinary ball, but it is called "cooperative charm ball" for today’s event. If you want to ask me what its charm is, let’s go to the event and look for it!

Rules of the game:

① The game is played between groups. The two students in the group are a partner. Both of them hold the ball with their backs and start from the starting point to the designed position at the other end of the classroom.

② During the march, put your hands on your chests and crossed. The ball cannot fall to the ground, and the hands can’t touch the ball, otherwise returns to start again.

③ After returning to the starting point, pass the ball by hand to the next pair of partners in the group. The team that finishes the relay first wins.

Note: Please discuss the secret of winning and practice for a while. At the end of this round, ask the students if they still want to play? This time it was replaced with a leg-side ball. Other rules and

requirements of the game are the same as the previous round. Now communicate with each other to learn the lessons of the previous round and practice a little.

Sharing: In these two rounds of events, we cooperated to complete the relay race. How do you feel about the cooperation? Does cooperation matter? What are the secrets of winning? Did some minor episodes appeared during the cooperation? How many failures have we encountered in our group? How did we succeed? Communicate in a circle in groups. Then the group sends representative of your group to share in the whole class.

Game 2: Competition in cooperation

Game: Now each column of classmates is a team, the teacher will show the first classmate a word, After the teacher announced the start, the first person must pass to the next one in turn until the last member of your team. The other players lie on the table, and Looking up when it reaches you. After the last one completes the task, please confirm how many groups are completed, and then make the corresponding number by hand.

Rule: Don't open your mouth during delivery, you can only express with your body above the shoulder. See which group is transmitted quickly and accurately.

Sharing: The fastest and most accurate group please send your little representative to share with you the secret of your victory.

(4) Summary of the activity

There are some requirements for good cooperation. It requires the students to constantly blend in with each other, the students need to work together and focus on cooperation. Only in this way can our cooperation be most effective and enjoyable.

2.6.4 Activity name: Know etiquette and be polite.

(1) Purpose of the activity:

✧ Learn to greet guests politely, answer the phone politely and unite your friends.

✧ Actively learn and use polite expressions to be polite in life.

(2) The record of materials

✓ 10 polite expression cards

✓ Courseware

(3) Descriptions of the activity

Game 1: Robbing music

Let's play a small game, "grab and grab", to see who answers quickly and accurately.

How do you say hello to others? Say hello softly, beckoning ...

Would you like to tell parents when go out in the morning?

What did you accidentally bump into your classmates during class.

What would you say with the help of others.

What do you need to say after classmates apologize to you.

What do you need to say when a guest arrives at home.

Game 2: Situational experience

Teacher: The classmates' responses were really wonderful, and I'm moved. We should not only show civility and politeness in words, but also put them into practice. It is regrettable that uncivilized phenomena often appear around our lives. Let's take a look at some pictures together. In the process of watching, you can tell what you see to your deskmate.

Teacher: Look, these uncivilized behaviors happen to us. In fact, we have all had uncivilized behaviors that are hard to tell, and sometimes make us embarrassed. Now let us bravely talk about things that we are usually uncivilized or impolite.

Teacher: It seems that in real life, there are quite a lot of uncivilized behaviors of our classmates. I believe that many of you have already recognized your shortcomings and can speak up bravely. For you brave!

Teacher: How do you plan to get rid of these uncivilized words and deeds? You can say it to yourself or others. Please speak freely.

(4) Summary of the activity

Here I sincerely hope that you can converge and correct some of the uncivilized behaviors through this class. You may not be able to change them for a while, but as long as you have the determination to change, you will do it. Not only do we have to work hard by ourselves, call on the people around us to work together. Everyone strives to be a polite pupil who understands etiquette.

2.7 Honesty and Shame

2.7.1 Activity name: Be an honest child.

(1) Purpose of the activity:

✧ After doing something wrong, students voluntarily admit their mistakes, treat others with sincerity, and do not lie.

✧ Promise others' things, be able to do what you say, and keep your promises.

(2) The record of materials

✓ Courseware

(3) Descriptions of the activity

Game 1: Inconsistent thinking and saying

Rules of the game: Two people at the same table play in a group. Each group will get a colored paper. The game is divided into two rounds: In the first round, choose one to say the color of these words in 1 minute, under the supervision of another person. If the pause time exceed 1 second, or there is an error, it's a foul. In the second round, the two exchanged roles.

Sharing: Students who successfully completed the task in these two rounds of games without fouling, please stand up. Let's applaud and encourage them.

What is the difficulty of this game? Let us talk about it, why do people make mistakes and foul?

How can the teacher change to make it easy for everyone to say these colors and not easy to make mistakes?

Summary: Is it easy to recognize if I matche the words with the corresponding colors?

These words are all learned by everyone and are engraved in the heart. It is more difficult when there is a conflict between what is said in the mouth and the appearance of the ideal of the heart. When you think about what this mouth says is consistent with your ideals, what kind of performance is this? I will say whatever the facts are, and it will not go against my wishes. What kind of performance is this? —— (This is an honest performance)

Game 2: Activity perception

Everyone, you see the general idea of the picture: After school, a boy went to play in the community and accidentally kicked the ball on the glass of the neighbor's house. What will he do? If it were you, what would you do? why?

Summary: Since we are sensible, parents and teachers have taught us to be an honest child and not to lie, because lying will lose the trust of others in you, and losing the trust of others will have serious consequences and even threaten people's lives. There are many stories about it.

Game 3: Role playing

One day, classmate Xiaozhen said that she would come to learn about the origami flower basket the next day, and Song Qingling agreed happily. The next morning, Song Qingling went to bed and was anxious to clean up the table, preparing colored paper and scissors for the origami basket, waiting for Xiaozhen 's arrival. Her mother said, "We are going to Uncle's house today. Haven't you always wanted to go? Let's teach Xiaozhen another day, okay?" Song Qingling shook her head: "No, I have an appointment with Xiaozhen." Father also came to persuade her: "You can teach another day!" Song Qingling resolutely said: "No! If you promise others, you must do it." Mom and Dad couldn't help Song Qingling's stubbornness, so she had to stay at home alone. Song Qingling waited for a long time, but Xiaozhen didn't come. She waited for until the afternoon, Xiaozhen still didn't come. In the evening, Mom and Dad came back and asked Song Qingling with concern: "Are you having fun?" Song Qingling said calmly: "Xiaozhen didn't come, she might have something else to do." Mom said regretfully: "I already knew, you just go to Uncle's house with us, "Song Qingling said:" Since I promised Xiaozhen, I have to wait for her. If Xiaozhen comes, I am not at home, and no one will believe me in the future! "

This is the story of Song Qingling. Whoever thinks she can paint the expressions of the characters at that time and the sound of voice performances?

Sharing:

a. Who did Song Qingling agree to? How did she do it?

b. How did Mom and Dad do it? What is Song Qingling's reaction? How do you feel?

c. What are the qualities of her worth learning from?

d. Song Qingling waited for a day, and Xiaozhen did not come. What do you think of this matter?

e. What kind of influence or consequence will Xiaozhen do in this way?

Summary: Being a person must keep the agreement, and failure to observe the agreement is a manifestation of untrustworthiness.

(4) Summary of the activity

You have gained a lot, and the teacher is really proud of you! We say that this seed of honesty has taken root and sprouted in our hearts. The team leader please take out the honest leaf in your hands. If you can be an honest person in your future life, just sign your name on this leaf, then the team leader posted on the tree. Teacher plays the song "The Real World". Look at the honest tree that was bare

just now, and now it has become leafy all at once because of our sincerity, kindness and beauty. May this honest little tree bloom in this and beautiful world. Finally, let us speak out our promises aloud: We have to be honest and be honest.

2.7.2 Activity name: Lying and honesty.

(1) Purpose of the activity:

✧ Let students know what is honesty.

✧ Students learn not to lie,

(2) The record of materials

✓ The video "Wolf Is Coming"

✓ Courseware

(3) Descriptions of the activity

Game 1: "Wolf is Coming"

Sharing:

A. The wolf really came and the little boy shouted that the wolf was coming, why no one believed him?

B. If the little monkey does not help the little boy, what will happen to him?

C. What are the consequences of dishonesty and deception? How do you feel after watching this video?

Summary: If we are dishonest and always like to deceive people, it is ourselves who are ultimately hurt.

Discussion on integrity

See the picture

① For those patients who are seriously ill, such as cancer, do doctors and family members have to tell the patients their true condition for integrity?

② In the picture, the aunt just wears a new dress today and asks you: Is it pretty? In fact, you feel bad, but in order to make your aunt happy, you say good-looking. You didn't tell the truth, so is it dishonesty?

③ Looking at this picture, who can tell what is said? Mom didn't tell the truth, is this dishonest?

④ For these three pictures, please discuss in the group for 3 minutes.

Sharing: As an elementary school student, the integrity to be done first is not to falsify, for example don't cheating in exams, don't conceal wrong things, etc. Any other examples? But is honesty to tell the truth everywhere? Explain your point.

Summary: The question of honesty and dishonesty cannot be measured simply by telling the truth or not, but by looking at what kind of situation and to whom. For example, if a doctor wants to make more money, he will say that a person who is not sick is sick, and a minor illness is said to be a serious illness.If only one medicine could cure the disease, but several medicines are prescribed, this is dishonesty. However, if you don't tell the actual condition of the critically ill patient in order to let the patient have no ideological pressure and get better treatment, it can't say that you are dishonest. This is white lie. As another example, my father was obviously very sick, but my mother told me that everything is fine. Mom doesn't want me to worry and worry about it. This is also a "white lie".

Game 2: Honest Story Meeting:

Sharing: There are many stories about honesty. Everyone knows honesty stories. They can be on the storybook or the real stories around you.

(Child holding an empty flower pot) After listening to this story, who would explain why did the king choose Xiongri as heir?

(The king believes that an honest child can govern the country. It can be seen that honesty is the most important thing.) Who will speak one more?

(Lenin breaks the vase) Lenin can be honest at a young age, which is really admirable. Who is talking about one?

(The story of the three axes) Although poor, he is not impressed by gold and silver, insisting on his own iron axe. He is truly honest.

(4) Summary of the activity

You guys are really good. I feel so proud and proud of having so many honest children in our class.

In order to reward you, I wrote a small poem specially for everyone.

Liars are harmful to yourself, to everyone

To be honest from an early age, everyone praises honest children.

The teacher asks everyone if they would like to be a student who jokes every day? Or are you willing to be an honest student? Now let us all say I am willing to be a good student with good faith" loudly. The louder the voice, the stronger your desire. Well, the teacher will say three times, 123 ...

2.7.3 Activity name: Trustworthy me.

(1) Purpose of the activity:

✧ Let students understand the role of trustworthiness.

✧ Students learn to keep their promises and do not lie.

(2) The record of materials

✓ Courseware

(3) Descriptions of the activity

Introduction of stories:

In ancient times, there was a person called Zeng Zi. One day his wife was going to the market, but the younger son was crying and going to go, so his wife coaxed him to come back and kill the pigs for him to eat. The son believed that it was true, so he wouldn't make trouble to go. The wife came back and saw that Zeng Zi was about to kill the pig and quickly stopped him. Zeng Zi said: "If the parents don't count if they speak, the child will learn to cheat and don't talk about credit. How will he gain a foothold in the world in the future?" Zeng Zi finally killed the pig.

Sharing: Please think about it, a pig was very expensive at that time, but Zeng Zi killed a pig in order to fulfill an inadvertent promise, why? Who would like to share your thoughts with your classmates?

Summary: Zeng Zi killed the pig and gave it to his son in order to educate his son that he should speak through his own behavior, and promise that others' things should be done. Zeng Zi set an example for his son, educating his son to be honest and trustworthy, otherwise it will be difficult to gain a foothold. From Zeng Zi's point of view, honesty is really very important. What is the importance of integrity? Let's learn "The Trustworthy Me" together today to explore the importance of honesty.

Game 1: The mouth and fingers are different

Rules of the game: The two are in a group, standing face to face, reaching out a hand.

After the game starts, the two together say that the mouth and fingers are different, and then say a number from 1 to 5, and at the same time, the finger should indicate a number from 1 to 5.

The number stated on the mouth is different from the number indicated by the finger, otherwise it will be punished. Instead, say "Sorry, I was wrong." loudly. The game continues.

You have 3 minutes to play this game, remember that you cannot always say the same number every round.

Sharing:

1) Are you having fun? How does everyone feel about this game? Why are you punished?

2) What does this game tell us?

3) What is the punishment and what do you understand from it?

Preset answer: It is difficult, laborious and error-prone when the number spoken by the mouth is different from the number made by the finger.

Be a good boy who is honest, does not falsify and keeps promises. The courage to say “Sorry, I’m wrong” and taking the initiative to admit mistakes is also a sign of integrity.

Summary: Being an honest and trustworthy person will make your life easy and comfortable, and honestly admit mistakes will make you live frankly and pleasantly.

Game 2: Cultivate the concept of integrity and make "integrity card"

Requirement: Make an "integrity card" with your own hands. Write whether you are an honest person in your life and from what can show dishonesty? Have you ever experienced dishonesty? After learning honesty and trustworthiness, what will you do in the future?

Note: Distribute a blank sheet of paper to everyone and ask students to write their own examples of integrity in life. Then the teacher asks the students to fold it and give it to him.

Comment: Through the integrity card, let students understand that they should be honest and trustworthy. We should despise false and untrustworthy behavior, and feel uneasy and guilty about our dishonest and untrustworthy behavior.

(4) Summary of the activity

We know that honesty and trustworthiness are very important for our society and for us. Without integrity, the entire society will lose its sense of security, and people will lose their minimum trust. It hurts everyone, including themselves. Therefore, as a primary school student in the new era, we can not only learn to be honest and trustworthy, but also contribute to the promotion of social integrity and virtue.

2.7.4 Activity Name: Speak with faith.

(1) Purpose of the activity:

✧ Let students understand the role of trustworthiness.

✧ Cultivate students' independent ability.

✧ Students learn to be trustworthy and independent.

(2) The record of materials

✓ Courseware

(3) Descriptions of the activity

Teacher: Are you familiar with this scene? (The teacher randomly asks several students to play the hook game.) Yes, it’s the hook game that we often play, and it represents a kind of commitment between us.

Students watch a group of pictures.

Teacher: In addition to the "pull hook" commitment, there are many forms of commitment in social life, such as post commitment, commercial commitment, government commitment, modern credit currency, etc. (The teacher shows the relevant pictures)

Group discussion:

Teacher: Invite students in a group of four to discuss: What is the importance of job commitment, business commitment, government commitment, modern credit currency, and "pull hook" commitment?

(Through discussion, students make it clear that no matter whether it is an individual, an enterprise, a bank or the government, without words, they will not be trusted by others. Having faith in words is the foundation of standing.)

Teacher: Right! Regardless of whether it is an individual, an enterprise, a bank or a government, only if they can be consistent at all times, treat others honestly, speak things and talk about credit, will they win the trust and respect of others.

Game 2: What do you do with such things in real life?

Case 1: Zhou Bing and Song Jie are good friends. They promised that no matter who is in trouble, they must help each other. One day Zhou Bing wanted to copy Song Jie's homework but he didn't agree. Zhou Bing said angrily: "You can’t help me at all, you really don’t talk about credit."

Discussion: What do you think of this matter?

Case 2: Li San was envious of seeing his classmates spend money generously. One day, taking advantage of his inattentiveness at the same table, he took 10 yuan from his classmates. When Teacher Zhang investigated, he didn't admit it at all. Then teacher Zhang said: "If you trust me, tell me the truth. I promise not to tell anyone." After listening to Teacher Zhang, he quickly told Teacher Zhang the truth.

Discussion: Do you know the reason?

Summary: Copying homework to a good friend is an act of non-responsibility. It is a good friend that hurts himself. It is also a manifestation of dishonesty. Although Li San is wrong to take other student’s money, he can take the courage to bear his own errors, which is actually an expression of integrity. So how can we keep our promise? The above examples require us to develop good quality of integrity, we should start to cultivate good quality of trustworthiness from an early age, usually we must strictly demand ourselves. We need to be consistent and faithful.

(4) Summary of the activity

We know that honesty and trustworthiness are very important for our society and for us. Without integrity, the entire society will lose its sense of security, and people will lose their minimum trust. It hurts everyone, including themselves. Therefore, as a primary school student in the new era, we can not only learn to be a trustworthy person, but also contribute to the promotion of social integrity and virtue.
